# Health Tract, No. 266

**Published:** 1867-03

**Authors:** 


HEALTH TRACT, No. 266.

### CLEANLINESS AND CHARACTER.

In the year 1840 a youth entered Milwaukie to make his fortune in the world, with five dollars and a quarter in his pocket, and a thousand miles from home, without a friend or even an acquaintance near him. He was a young lawyer and as it was two months before the opening of the court, and sooner than that he had no means of replenishing his purse, he frankly stated to his landlord his financial situation and asked credit for his board, which was granted him. He then spent his five dollars in having his clothing washed, and one or two other items, and entered upon the great work of life. But finding his money all gone, and hearing that an old schoolmate of his lived in that part of the country, he walked fifty-six miles to borrow from him ten dollars. He returned to town, was admitted to the bar on the opening of the court and soon fought his way into an excellent practice, in the prosecution of which he traveled over a great portion of the states on foot and on horseback.

There was something in that young man, who among perfect strangers in the far West, would expend nearly all of the last five dollars he had in the world, in having his stock of clothing washed and repaired. There, too, was self-respect and a consciousness of personal elevation. But the next step was akin to it; the frankness and honesty of making a plain statement to his landlord at the ontset, of his means, his plans and his hopes. Nine men out of ten perhaps, would have worn the soiled clothing longer and would have kept the scantiness of funds a profound secret, hoping that before they were exhausted something would turn up to improve the finances, shutting the eyes to the fact that in the event of a failure in this direction, a confiding landlord would be the loser. This is the very rock on which thousands founder every year, risking the money of the friends who have reposed confidence in them. The man who ever does that is always a scoundrel at the core. You may risk what really belongs to you but the means of another, never.

That tidy, self-respecting, honorable young lawyer, has, at the end of a quarter of a century, become one of the constitutional advisors of the President of the United States. Physical cleanliness and moral purity and elevation of character have a close connection; while tidiness in dress has a strong alliance to strict justness and fitness of action. The accomplished Bostonion, Washington Alston, suddenly remembering that he had a hole in his stocking, declined entering a private mansion where there was a party. "They don't know it," said his friend. "But I do," said the great artist and turned away. Cleanliness of person promotes health of body and this in turn naturally begets purity of mind and moral elevation. Such persons are quite as much concerned in having the inner and unseen as tidy and as clean as the outer and the visible; they are pure from principle, not policy; pure from the love of it, not from man, for it is "the pure in heart" who "shall see God." All are robed in spotless white who stand before the "Great King."

